# Screening of differentially expressed genes associated with non-union skeletal fractures and analysis with a DNA microarray

**DOI:** 10.3892/etm.2014.1478

**Published:** 2014-01-08

**Authors:** JIAMING XU, CHANGQING ZHANG, WENQI SONG

**Affiliations:** Department of Orthopedics, Shanghai Sixth People’s Hospital Affiliated to Shanghai Jiao Tong University, Xuhui, Shanghai 200233, P.R. China

**Keywords:** non-union skeletal fractures, differentially expressed gene, interaction network, function enrichment analysis, pathway analysis

## Abstract

The purpose of this study was to identify the feature genes that are associated with non-union skeletal fractures using samples of normal union and non-union skeletal fracture microarray data. The gene expression profile GSE494 was downloaded from the Gene Expression Omnibus database and included 12 samples based on three different platforms (GPL92, GPL93 and GPL8300). Each of the platforms had four sets of expression data, two from normal union skeletal fracture samples and two from non-union skeletal fracture samples. The differentially expressed genes within the three platforms of expression data were identified using packages in R language and the differentially expressed genes common to the three platforms were selected. The selected common differentially expressed genes were further analyzed using bioinformatic methods. The software HitPredict was used to search interactions of the common differentially expressed genes and then FuncAssociate was used to conduct a functional analysis of the genes in the interaction network. Further, the associated pathways were identified using the software WebGestalt. Under the three different platforms, GPL92, GPL93 and GPL8300, the numbers of differentially expressed genes identified were 531, 418 and 914, respectively. The common gene CLU and its interacting genes were most significantly associated with the regulation of sterol transport and the osteoclast differentiation pathway. Upregulation of the gene CLU was identified by comparing data for normal union and non-union skeletal fracture samples. According to the function of CLU and its interacting genes, it was concluded that they inhibit the normal healing process following a fracture, and result in non-union skeletal fractures through the regulation of sterol transport and the pathways of differentiation in osteoclasts.

## Introduction

There are >15 million fractures treated in the United States annually and many more worldwide (1). While the vast majority of these fractures heal with appropriate orthopedic management, 10–15% of patients suffer complications that result in delayed- or non-union (2). Fracture healing is a multistage repair process that involves complex yet well-established steps that are initiated in response to injury, resulting in the repair and restoration of function (3). Numerous factors have been associated with failure of normal fracture healing, including the fracture location, the extent of soft tissue damage and interposition, the degree of bone loss in anatomic criteria, infection, inadequate reduction, poor stabilization/fixation factors that are exacerbated by treatment, patient characteristics, comorbidities and drug use (2).

Fracture repair involves the pathway of normal embryonic development, which consists of several cell types originating from the cortex, periosteum, surrounding soft tissue and bone marrow space (4,5). Different biological factors, which include recruitment, proliferation and differentiation of cell types, vascular regeneration, expression of growth factors (e.g. IGF, TGF-β and BMP) and appropriate biomechanical conditions, have been considered to be critical for the healing of bone fractures. Local imbalances of these different factors during conservative or operative fracture treatment may lead to delay of fracture healing or to fracture non-union (6). According to radiological and histological criteria, non-unions are generally classified into three types (7). Hypertrophic non-unions are often linked with insufficient fracture stability and appear to have an adequate blood, oxygen and nutrient supply, while atrophic non-unions are generally poorly vascularized (7). In defect non-unions, the fracture healing is affected by a lack of contact among fracture fragments (6).

Although clinical experience in the treatment of fracture non-unions is quite extensive, studies concerning the high-throughput screening and function identification of differential gene expression associated with fracture non-union are limited. The objective of this study was to document the feature genes and their interacting genes, also further explore their potential functions associated with non-union fractures.

## Materials and methods

### Affymetrix microarray data

The gene chip GSE494 was downloaded from the gene expression database Gene Expression Omnibus (http://www.ncbi.nlm.nih.gov/geo/) and is based on three platforms: GPL92, [HG_U95B] Affymetrix Human Genome U95B Array; GPL93, [HG_U95C] Affymetrix Human Genome U95C Array; and GPL8300, [HG_U95Av2] Affymetrix Human Genome U95 Version 2 Array. There were data for 12 bone samples of fractures in total in the three platforms, with each platform containing data for two normal healing fracture samples and two non-union fracture samples. All the original files and the platform probe annotation information files were also downloaded

### Data preprocessing and gene differences analysis

The original data were preprocessed using the R language Affy software package (8,9). The R language limma package (http://www.r-project.org/) was used to analyze for differentially expressed genes between all the normal and non-union samples (10), and Bayesian methods were used to conduct multiple testing correction. The threshold values were set as P<0.05 and | logFC |>1.

### Predicting the interactions of differentially expressed genes

A single gene is not able to regulate function; only protein-protein interactions (PPIs) have been marked as the main actors for all of the processes taking place in a cell and therefore great efforts have been focused towards understanding their biological function (11). Hence, the software HitPredict (http://hintdb.hgc.jp/htp/) was used to analyze the interactions between the differentially expressed genes (12,13). The HitPredict database was built by collecting information from the IntAct BIOGRID and HPRD databases through high-throughput or small-scale experiments of protein interaction associations and, according to the interaction score, estimating protein interactions (the interaction score is obtained according to a likelihood algorithm which uses a Bayesian network combining binding sequence, structure and functional annotations of the PPI in the calculation) (13). HitPredict collects PPI data from high-throughput, small-scale experiments, and considers a likelihood ratio of the resulting score of >1 as a high degree of confidence interaction (12). It has collected 239,584 PPIs from nine species, including humans and mice, of which 168,458 are predicted to have a high degree of confidence. The high degree of confidence interactions from the database were used in this study (experimental and likelihood ratio >1) to analyze the differential gene product.

### Enrichment analysis of genes

Differentially expressing genes were screened using enrichment analysis based on the hypergeometric distribution algorithm of FuncAssociate (14). The threshold value of P<0.05 was selected.

### Analysis for pathways involving genes in the interaction network

Proteins in a PPI network and the same module usually complete the same biological processes and functions by co-expression. In the present study, enrichment analysis using WebGestalt (15,16), which is based on the hypergeometric distribution algorithm, was used to analyze the pathway of interaction networks involving the differentially expressed gene and its interactions (P<0.05).

## Results

### Screening for differentially expressed genes

**Figure 1:**
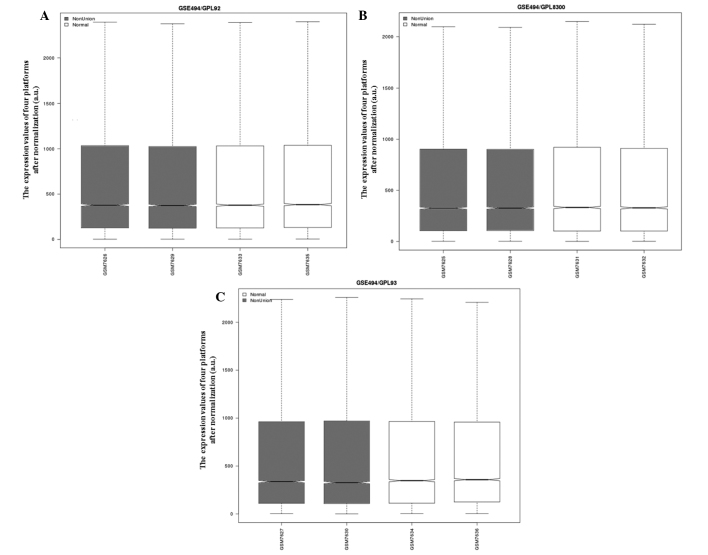
Normalized expression values data box plots. Expression data of the three platforms: (A) GPL92; (B) GPL8300; and (C) GPL93. The gray boxes indicate a non-union fracture sample of the platform, while the white ones indicate normal healing fracture samples. The box in the black line is the median of each set of data, which determines the degree of standardization of data through its distribution. The black lines in the boxes are almost in the same straight line, indicating a good degree of standardization.

**Figure 2:**
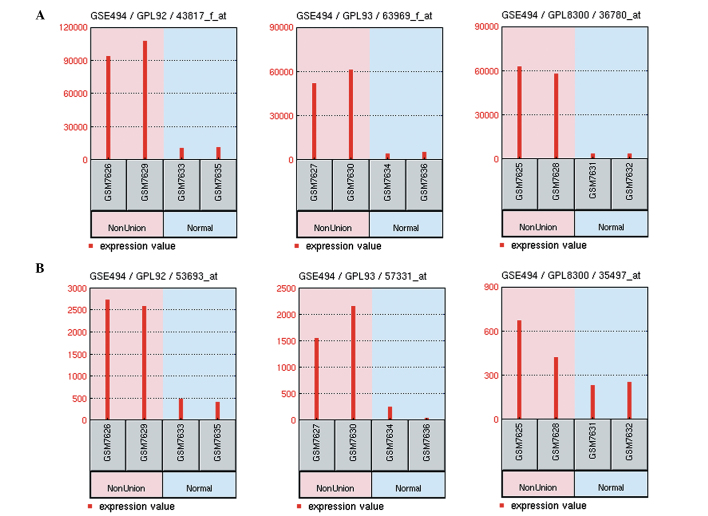
Expression data of the two differentially expressed genes. The expression data of (A) CLU and (B) TSPAN2 in the three platforms. The x-axis indicates grouping and the y-axis demonstrates expression data (a.u.). The height of the red column represents the expression value.

The differences between the normalized expression data were compared following data preprocessing (Fig. 1). A total of 531, 418 and 914 genes from the three platforms that met the difference threshold (P<0.05 and | logFC | >1) were screened. Two genes, CLU and TSPAN2, were identified to be the commonly differentially expressed genes in the three platforms. The expression values of these two genes were upregulated in the delayed healing fracture sample (Fig. 2).

### Predicting the interactions of the differentially expressed genes

**Figure 3:**
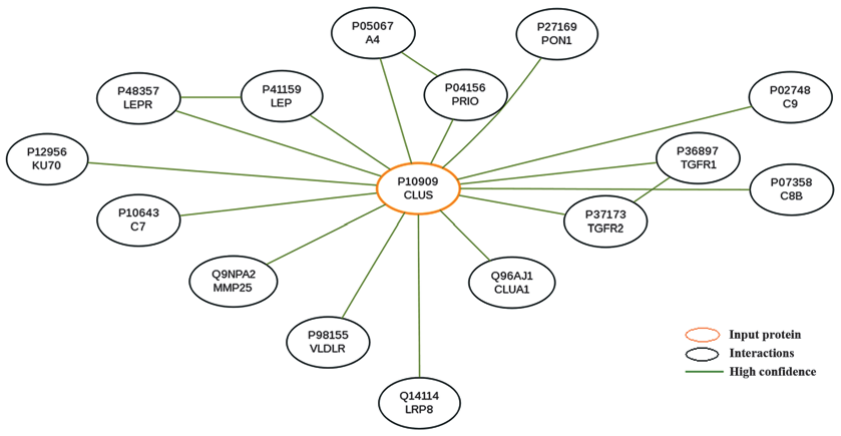
Co-expression network of CLU. The orange circle indicates the input gene CLU, and the black circles indicate interaction objects.

Using the software HitPredict to screen for all differentially expressed genes and their product interactions, the gene CLU and 44 interaction objects were obtained (Fig. 3; there were no interaction records of TSPAN2 recorded in HitPredict). The interaction objects of CLU and their likelihood of interaction scores are listed in Table I.

### Enrichment analysis of network genes

Enrichment analysis based on the hypergeometric distribution algorithm was conducted using FuncAssociate. A threshold value of P<0.05 was selected. As presented in Table II, five significantly enriched features were obtained. The most significantly enriched genes in the network were associated with sterol transport.

### Analyzing gene functions in the co-expression interaction network

By using WebGestalt, which is based on the hypergeometric distribution algorithm, to analyze the pathway of the interaction network involving the differentially expressed gene and its interactors (threshold value P<0.05), four pathways with significantly enriched genes were identified (Table III). One of the most significant pathways was osteoclast differentiation, which involved the SYVN1, MDM2, KEAP1 and CLU genes.

## Discussion

Non-union of a fracture is defined as the cessation of all reparative processes of healing without bone-union (17). As previous data has demonstrated that the diagnosis of non-union fractures is based on clinical symptoms and physical findings, including pain at the fracture site and evidence of pathologic motion (18), there are seldom studies on the high-throughput screening and function identification of differential gene expression in fracture non-unions. In the present study, the upregulated gene CLU and its 44 interaction objects were selected from a microarray chip composed of normal union and non-union skeletal fracture samples. According to the function of CLU and its interacting genes, the conclusion was reached that by inhibiting the normal healing process following a fracture, the selected genes regulated the healing of non-union skeletal fractures through participating in sterol transport and the pathway involved in the differentiation of osteoclasts.

CLU (also known as clusterin, apolipoprotein J, TRPM-2 and SGP-2) is highly conserved in different species, with approximately 70–80% protein homology in mammals with other species. CLU consists of a 449-amino-acid primary polypeptide chain. By its disulfide bridges, human CLU is cleaved into α and β chains (19). CLU expression is low in normal conditions but is induced by stress stimuli, suggesting that its function may be directly or indirectly associated with the stress response (20). In a number of studies, CLU has been demonstrated to be antiapoptotic, protecting cells against a variety of death signals (21–23). Although there have been no direct studies indicating that CLU is associated with fracture healing, a recent study has demonstrated that the mRNA levels of CLU are increased in early osteoarthritic articular (OA) cartilage, while they are decreased in advanced OA (24).IL-1α-stimulated cartilage explants have been demonstrated to produce decreased levels of CLU compared with those in untreated cartilage (25) and treatment with IL-1β also decreases the levels of CLU (26). Synovial apoptosis inhibitor 1 (SYVN1), also known as DER3 and HRD1, is an E3 ubiquitin ligase that is implicated in endoplasmic reticulum-associated degradation (27). It is cloned from rheumatoid synovial cells and is highly expressed in the synoviocytes of patients with rheumatoid arthritis (RA). Through its antiapoptotic effect, SYVN1 promotes the overproliferation of synoviocytes (28,29). Murine double minute 2 (mdm2) was first identified as the gene responsible for the spontaneous transformation of 3T3 cells (30). As an E3 ubiquitin ligase, mdm2 is a critical negative regulator of p53 by targeting it for ubiquitination and proteasomal degradation (31). Individuals carrying the mdm2 SNP309 T/G or G/G have been identified to exhibit a significantly earlier age of onset for osteosarcoma (32). In RA patients, the frequencies of the mdm2 SNP309 are significantly reduced (33), while the mdm2 SNP 309G/G is associated with higher levels of apoptotic activity in RA-derived synoviocytes (34). Kelch-like ECH associated protein 1 (Keap1) is a stress sensor and an adaptor component of Cullin 3-based E3 ubiquitin ligase (35). Under normal (unstressed) conditions, Keap1 activates and rapidly degrades Nrf2 through the proteasome pathway. Upon cellular exposure, as an E3 ubiquitin ligase component, Keap1 is inhibited, which provokes Nrf2 stabilization (36). A study has reported that the Nrf2-Keap1 signaling cascade is conserved in human skeletal muscle (37).

Fracture healing is a complex process that involves osteoblasts, osteoclasts and a variety of other cells and cytokines (38), which means it may be the outcome of the interaction of multiple genes. Although there have been only indirect studies that have indicating that CLU and its interacting genes are involved in healing fractures, there is significant evidence that they participate in the healing process. In conclusion, the generally stronger inhibition of osteoblasts in non-union fractures (39) combined with the results of the present study indicating that CLU and its interacting genes SYVN1, MDM2 and KEAP1 participate in the osteoclast differentiation pathway suggest that all the genes which were identified by screening may regulate the healing of fractures through an involvement in osteoclast differentiation.

## Figures and Tables

**Table I:** Genes interacting with CLU.

Interactors	Likelihood of interaction
TGFR2	999
KU70	999
TGFR1	999
PON1	999
PRIO	999
VLDLR	999
C7	999
LRP8	999
MMP25	999
A4	999
C8B	999
LEPR	999
CLUA1	999
C9	999
LEP	999
COMD1	999
LRP2	8.68
DISC1	3.37
TNIK	3.37
APOA1	3.37
GRB2	3.37
H2AX	3.37
NR4A1	3.37
FOS	3.37
MDM2	3.37
GCR	3.37
PPARG	3.37
ZNF24	3.37
B2CL1	3.37
MK09	3.37
RL23	3.37
BAT3	3.37
CYP2E1	3.37
RBBP8	3.37
KLF11	3.37
KEAP1	3.37
T22D4	3.37
SYVN1	3.37
UBC	3.37
NFKB1	3.37
IKBA	3.37
CUL1	3.37
FBW1A	3.37
RAD21	3.37

**Table II:** List of functions associated with enriched genes in the network.

ID	Term	P-value	Genes
GO:0032371	Regulation of sterol transport	0.00000703	LEP, APOA1, PON1, NFKB1, CLU
GO:0044421	Extracellular region part	0.0000341	LEP, C8B, APOA1, LEPR, CLU, PON1, LRP8, LRP2, MMP25, VLDLR
GO:0005576	Extracellular region	0.000481	LEP, C8B, C7, APOA1, C9, LEPR, CLU, PON1, LRP8, LRP2, MMP25, VLDLR
GO:0043233	Organelle lumen	0.004086798	FOS, APOA1, SYVN1, CLU, UBC, NR4A1, MDM2, NFKB1, KEAP1, CUL1
GO:0031974	Membrane-enclosed lumen	0.004673635	FOS, APOA1, SYVN1, CLU, UBC, NR4A1, MDM2, NFKB1, KEAP1, CUL1

**Table III:** List of pathways associated with enriched genes in the network.

ID	Term	P-value	Genes
hsa04380	Osteoclast differentiation	0.011605668	SYVN1, MDM2, KEAP1, CUL
hsa04920	Adipocytokine signaling pathway	0.022842413	LEP, LEPR, NFKB1
hsa04610	Complement and coagulation cascades	0.024136338	C8B, C7, C9
hsa04662	B cell receptor signaling pathway	0.028195492	FOS, GRB2, NFKB1
